# Comparative Transcriptome Analysis Reveals Early Pregnancy-Specific Genes Expressed in Peripheral Blood of Pregnant Sows

**DOI:** 10.1371/journal.pone.0114036

**Published:** 2014-12-05

**Authors:** Junye Shen, Chuanli Zhou, Shien Zhu, Wenqing Shi, Maishun Hu, Xiangwei Fu, Chuduan Wang, Yachun Wang, Qin Zhang, Ying Yu

**Affiliations:** 1 Key Laboratory of Animal Genetics, Breeding and Reproduction, Ministry of Agricultural, National Engineering Laboratory for Animal Breeding, College of Animal Science and Technology, China Agricultural University, Beijing, P.R. China; 2 Department of Animal Genetics, Breeding and Reproduction, College of Animal Science and Technology, China Agricultural University, Beijing, P.R. China; 3 Animal Husbandry and Veterinary Station of Beijing, Beijing, P.R. China; Huazhong Agricultural University, China

## Abstract

Early and accurate diagnosis of pregnancy is important for effective management of an economical pig farm. Besides the currently available methods used in early diagnosis of sows, circulating nucleic acids in peripheral blood may contain some early pregnancy-specific molecular markers. For the first time, microarray analysis of peripheral blood from pregnant sows versus non-pregnant sows identified 127 up-regulated and 56 down-regulated genes at day 14 post-insemination. Gene Ontology annotation grouped the total differently expressed genes into 3 significantly enriched terms, cell surface receptor linked signal transduction, G-protein coupled receptor protein signaling pathway and regulation of vesicle-mediated transport. Signaling pathway analysis revealed the only one significantly changed pathway was arachidonic acid metabolism. Of the differently expressed genes, nine (including *LPAR3*, *RXFP4*, *GALP*, *CBR1*, *CBR2*, *GPX6*, *USP18*, *LHB* and *NR5A1*) were found to exert function related to early pregnancy processes. This study provides a clue that differentially abundant RNAs in maternal peripheral blood can help to identify the molecular markers of early pregnancy in pigs.

## Introduction

Successful implantation in mammalian relies on an intricate discourse between the blastocyst and the maternal uterus [1]. The reciprocal and primary dialogue essential for the implantation process involves not only gonadal steroids but also other biologic molecules, which broadly include growth factors, adhesion molecules, chemokines and cytokines [2]. The cellular events that occurred in the endometrium throughout the various stages of implantation in swine have been explored and described [3], [4].

Embryo implantation is the first and most important stage of pregnancy; in swine this occurs at Days 13–14 of gestation [5]. For economic reasons, early and accurate pregnancy diagnosis is essential in domestic animal (such as swine and bovine) management. The diagnosis of pregnancy has been sought since long by farmers, which mainly includes visual, clinical and laboratory methods [6]. Heretofore, the techniques used for the pregnancy detection in sows are mostly based on physiological or behavioral changes that normally occur following conception, with having their advantages and limits, respectively [7].

Once implantation is complete, the maternal blood is brought into contact with the developing embryonic blood vessels. In ruminants, such as cattle and sheep, interferon-tau (IFNT) secreted by trophectoderm is the pregnancy recognition signal [8]. Experimental evidence showed that IFNT regulates expression of genes in peripheral blood leukocytes (PBLs) during early pregnancy of ewes [9]. Study on dairy cattle showed that mRNA expression levels of IFNT stimulated genes (eg. *ISG-15*, *Mx1* and *Mx2*) were significantly increased in pregnant compared with bred, non-pregnant cows during early pregnancy in PBL [10]. In humans, a number of investigators have discovered circulating fetal nucleic acid, including DNA and RNA, in the maternal plasma decades ago [11], [12]. Fetal DNA can be isolated from maternal blood as early as the fifth postmenstrual week [13] and fetal RNA has been detected as early as the sixth postmenstrual week [14]. Since then, circulating fetal nucleic acids have opened up an approach for noninvasive prenatal diagnostics and have been widely analyzed in scientific and clinical fields [15]. Interestingly, Mayer *et al.* [16] have recently reported that circulating fetal nucleic acid can be used as biomarkers for the detection of early pregnancy in cattle. These studies may imply that maternal peripheral blood can be used for an early pregnancy diagnosis in domestic animals, such as swine.

To date, the molecular cascades in peripheral blood of early pregnancy sows have not been elucidated. Hence, detection and investigation of biochemical markers in maternal peripheral blood at the early stage of implantation will likely contribute to a better understanding of the regulation of implantation and to the early detection of pregnancy in the near future. In this study, we aimed to compare global gene expression profiles of the maternal peripheral blood in pregnant sows with the non-pregnant sows at 14 days after insemination.

## Materials and Methods

### Ethics Statement

Animal experiments were approved by the Animal Care and Use Committee of China Agricultural University and the experiment was performed according to regulations and guidelines established by this committee.

### Animals and Tissue Collection

A total of 45 healthy Yorkshire sows (4th–6th parity) were collected from the breeding population based on the results of their breeding soundness examination. During estrus, all sows were artificially inseminated twice at an interval of 12h with extended semen from the same Yorkshire boar. Pregnancy diagnosis was conducted using real-time B-mode ultrasound scanner at day 35 post insemination [17], and the sows were subsequently divided into pregnancy and non-pregnancy groups. To minimize the adverse effects of genetic heterogeneity in microarray experiments, three half-sib pairs and one unrelated pair were selected with four sows from the pregnancy group and the other four from the non-pregnancy group.

Peripheral blood sample was collected at 14 days after insemination from the submandibular vein of each sow, thoroughly mixed with the cell lysate (Bioteke, Beijing, China) within 20 min, and stored at −70°C until further processing.

### Total RNA Isolation

Total RNA was isolated from peripheral blood samples using blood total RNA fast extraction kit (Bioteke, Cat No. RP4001) as described in File S1 and column purified using an RNeasy Kit (QIAGEN, Cat No. 74104) according to the protocols. RNA quantity was measured by Nanodrop ND-1000 spectrophotometer (Thermo Scientific), and RNA quality was further assessed by calculating RIN (RNA integrity number) with an Agilent Bioanalyzer 2100 (Agilent Technologies, Santa Clara, CA, USA). RNA samples that passed the quality control metrics, *i.e.* 2100 RIN > = 7.0 and 28S/18S >0.7, were sent for microarray analysis.

### Microarray Hybridization and Data Normalization

Microarray hybridization was performed according to the protocols of Agilent porcine 4×44 k one-color GeneChip. Total RNA was amplified and labeled by Low Input Quick Amp Labeling Kit, One-Color (Cat No. 5190-2305, Agilent technologies, Santa Clara, CA, US), following the manufacturer's instructions. Labeled cRNA were purified by RNeasy mini kit (Cat No. 74106, QIAGEN, GmBH, Germany). Each slide was hybridized with 1.65 µg Cy3-labeled cRNA using the Gene Expression Hybridization Kit (Cat No. 5188-5242, Agilent technologies, Santa Clara, CA, US) in Hybridization Oven (Cat No. G2545A, Agilent technologies, Santa Clara, CA, US) at 65°C, 10 rpm, according to the manufacturer's instructions. After a 17-hour hybridization, slides were washed in staining dishes (Cat No. 121, Thermo Shandon, Waltham, MA, US) with Gene Expression Wash Buffer Kit (Cat No. 5188-5327, Agilent technologies, Santa Clara, CA, US) following the recommendation of the manufacturer.

The washed slides were scanned by Agilent Microarray Scanner (Cat No. G2565CA, Agilent technologies, Santa Clara, CA, US) with default settings, Dye channel: Green, Scan resolution  = 5 µm, PMT 100%, 10%, 16 bit. Raw data was acquired by Feature Extraction software 10.7 (Agilent technologies, Santa Clara, CA, US) and normalized by Quantile algorithm, Gene Spring Software 11.0 (Agilent technologies, Santa Clara, CA, USA).

### Microarray Data Analysis

The normalized data were analyzed using GeneSpring software version 11.0 (Agilent Technologies) to screen differently expressed genes with criteria of *P*<0.05 and |FC| >1.5 (NCBI Accession No. GSE61986). Positive FC-value means that the gene was highly expressed in peripheral blood of pregnant sows, while negative FC-value means that the gene was highly expressed in peripheral blood of non-pregnant sows. To annotate the biological function of the differentially expressed genes in peripheral blood between pregnant and non-pregnant sows, DAVID (Database for Annotation, Visualization and Integrated Discovery) [18] was used to perform GO and KEGG pathway analysis.

### Quantitative Real-Time RT-PCR (qRT-PCR)

Samples of total RNA isolated and purified as described previously were reverse transcribed using High Capacity cDNA Archive Kit (ABI, USA). The reverse transcription reaction was incubated at 25°C for 10 min, 37°C for 120 min and 85°C for 5 min. The cDNA samples were then analyzed with real time RT-PCR using a LightCycler 480 Real-Time PCR System (Roche, Hercules, CA, USA). Seven genes were selected to perform qRT-PCR using intron-spanning primers (sequences are shown in Table 1). The real time RT-PCR reactions were performed using SYBR Green PCR Master Mix (Roche, Switzerland) in 20 µl reaction volumes containing 10 µl SYBR Green Master Mix, 5.5 µl nuclease free water, 1.0 µl of each primer, and 2.5 µl diluted cDNA. The pig housekeeping gene glyceraldehyde-3-phosphate dehydrogenase (*GAPDH*) was used as an internal standard to normalize cDNA input, since its expression was not changed across the two groups according to the microarray analysis. Triplicate qRT-PCRs were performed on each cDNA and the average Ct was used for further analysis. The relative quantification of gene expression was calculated using the 2^−ΔΔCT^ method.

**Table 1:** Primers for quantitative real time PCR and RT-PCR.

Genes	Genbank Accession NO.	Primers	Sequence	Product length (bp)
*CHGB*	NM_214081	F	*5′-AGCCTTGAGGTGGACAAACGA-3′*	182
		R	*5′-TGGGTAGGATGGCGGGTTC-3′*	
*USP18*	NM_213826	F	*5′-GGGCTCTTCCTCCGTCTCA-3′*	111
		R	*5′-CACCTCATGCGGTTCTCCA-3′*	
*VWF*	AF052036	F	*5′-TTCTGAAGAGTGCCTCGGTGTT-3′*	186
		R	*5′-TGTGGTCCATCCAGCCGTAGA-3′*	
*LPAR3*	NM_001162402	F	*5′-CTGGGTGATCGCCATCTT-3′*	108
		R	*5′-AGGTAACTCCTGCTGTAAACG-3′*	
*NR5A1*	NM_214179	F	*5′-TGGTCAAGGGCACCCGTTAG-3′*	97
		R	*5′-ACAGGGACAGAGTCAGCAAGGA-3′*	
*PPARD*	NM_001130241	F	*5′-TAAGGATGGGCTGCTGGTGG-3′*	174
		R	*5′-AGAATGATGGCTGCGATGAAG-3′*	
*BIN1*	NM_001097440	F	*5′-GACTGGAACCAGCACAAGGA-3′*	186
		R	*5′-TTACAAACGAAAACAGGAGGAA-3′*	
*GAPDH*	NM_001206359	F	*5′-TTTGGCTACAGCAACAGGGTG-3′*	188
		R	*5′-TCTGGGATGGAAACTGGAAGT- 3′*	

## Results

### Identification of Differentially Expressed Genes in Peripheral Blood of Pregnant and Non-pregnant Sows

**Figure 1:**
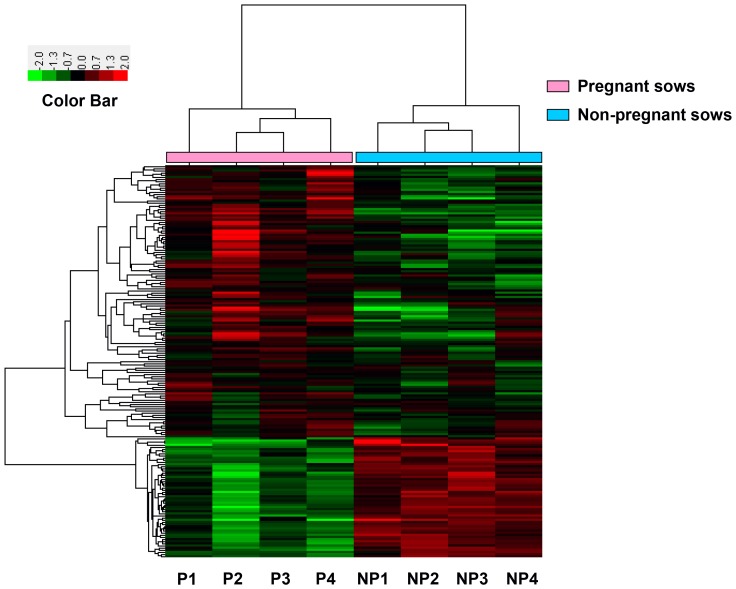
A heatmap of differentially expressed genes between the pregnant and non-pregnant sows. Red and green represent an increase and decrease in the gene expression levels, respectively, compared between 4 pregnant sows and 4 non-pregnant sows. Fold change > = 1.5 and *P*<0.05.

A total of 183 transcript-based probe sets were identified differentially expressed between the pregnancy (*n* = 4) and non-pregnancy (*n* = 4) sows at day 14 post-insemination under the criteria of FC > = 1.5 and *P*<0.05. The heatmap of the 183 differentially expressed genes was shown in Figure 1. Among the 183 transcripts, 127 were expressed at higher levels in pregnant sows (referred to hereafter as up-regulated genes) compared to non-pregnant sows (Table S1), while the other 56 transcripts were detected with lower expression in the pregnant animals (referred to hereafter as down-regulated genes) (Table S2).

### GO Term and KEGG Pathway Annotation of the Differentially Expressed Genes

To fully inspect the function of the differentially expressed genes in peripheral blood between the pregnancy and non-pregnancy sows, we performed a GO term and KEGG pathway annotation for the 183 differentially expressed genes using the DAVID gene annotation tool (HTUhttp://david.abcc.ncifcrf.gov/UTH) [19]. Three categories are included in GO: cellular component, biological process and molecular function. GO term annotation results showed that cell surface receptor linked signal transduction, G-protein coupled receptor protein signaling pathway and regulation of vesicle-mediated transport are the significantly enriched GO terms (*P*-value <0.05). Besides, there are 7 GO terms whose enrichment *P*-values are not significant at the 0.05 level also having been summarized in Table 2. For the 183 differentially expressed genes, the only significantly enriched pathway with the *P*-value <0.05 was arachidonic acid metabolism, which was also listed in Table 1.

**Table 2:** GO functional enrichment of the differentially expressed genes.

GO Term	Count	Genes	Fold enrichment	*P*-Value
GO:0007166∼cell surface receptor linked signal transduction	7	*LPAR3, RXFP4, GALP, S1PR5, LOC396643, SMAD3, P2RY2*	2.68	0.0323
GO:0007186∼G-protein coupled receptor protein signaling pathway	6	*LPAR3, RXFP4, GALP, S1PR5, LOC396643, P2RY2*	3.08	0.0336
GO:0060627∼regulation of vesicle-mediated transport	2	*BIN1,RAB3A*	38.00	0.0497
GO:0004090∼carbonyl reductase (NADPH) activity	2	*CBR1, CBR2*	37.36	0.0508
GO:0001619∼lysosphingolipid and lysophosphatidic acid receptor activity	2	*LPAR3, S1PR5*	37.36	0.0508
GO:0006887∼exocytosis	2	*CHGB, RAB3A*	22.80	0.0815
GO:0004221∼ubiquitin thiolesterase activity	2	*USP18, UCHL1*	22.41	0.0832
GO:0008289∼lipid binding	4	*ALB, LOC396596, PLTP, NR5A1*	3.56	0.0910
GO:0032940∼secretion by cell	2	*CHGB, RAB3A*	19.00	0.0971
GO:0046903∼secretion	2	*CHGB, RAB3A*	19.00	0.0971
ssc00590: Arachidonic acid metabolism	3	*CBR1, CBR2, GPX6*	8.18	0.0467

### Identification of Putative Molecular Markers of Sow Early Pregnancy in Maternal Peripheral Blood

The identified differentially expressed genes in this study were compared to one study of transcriptome changes in the endometrium of pregnant sows [3]. Due to the fact that different tissues were tested, only one common gene (i.e. lysozyme, *LYZ*) was identified in both studies. Nevertheless, homologous members of several gene families, including *AQP10/AQP11*, *F7/F5*, *GPX6/GPX3* and *GPR56/GPR68*, were identified in our study and in the previous study, respectively, which indicated that the same pathway may be exerted during early pregnancy in pig.

Furthermore, the genes revealed by the present study were illustrated by some related studies in other species, including cattle and sheep which show a non-invasive type of embryo implantation. At least nine genes (Table 3) were found to be associated with the physiological or biochemical process during early pregnancy.

**Table 3:** The mRNA expression changes of predicted molecular markers during early pregnancy in sow peripheral blood.

Genes	Probe Name in Microarray	Genbank ID	Mean fold change by Microarray (Pregnant/Non-pregnant)
*LPAR3*	A_72_P406683	NM_001162402	4.12
*RXFP4*	A_72_P177901	NM_001005844	2.51
*GALP*	A_72_P442900	NM_213825	2.20
*CBR2*	A_72_P655824	NM_213827	1.59
*CBR1*	A_72_P146631	NM_214073	−1.50
*GPX6*	A_72_P476541	NM_001137607	2.11
*USP18*	A_72_P417087	NM_213826	1.84
*LHB*	A_72_P651922	NM_214080	1.72
*NR5A1*	A_72_P444602	NM_214179	5.55

### Validation of the Microarray Results by qRT-PCR

To verify the microarray results, seven differentially expressed genes were selected to perform qRT-PCR using intron-spanning primers. Among them, five genes were up-regulated expressed in pregnancy sows and the other two were down-regulated. As shown in Table 4, the expression profiles of these genes detected by qRT-PCR were consistent with those by microarray, which confirmed the reliability of our microarray data. QRT-PCR detection tended to display a higher fold change than microarray, especially for those highly expressed genes, which may result from the more specificity of qRT-PCR [20].

**Table 4:** Validation of microarray results by real-time quantitative PCR.

Genes	Probe Name in Microarray	Mean fold change by Microarray (Pregnant/Non-pregnant)	Mean fold change by Q-PCR (Pregnant/Non-pregnant)
*CHGB*	A_72_P658293	3.50*	9.17*
*USP18*	A_72_P417087	1.84*	3.32*
*VWF*	A_72_P293294	−1.85*	−3.84*
*LPAR3*	A_72_P406683	4.12**	6.72**
*NR5A1*	A_72_P444602	5.55**	12.74*
*PPARD*	A_72_P165296	3.47*	3.11*
*BIN1*	A_72_P672677	−1.69*	−3.11*

The numbers here represent the fold change of gene expression in pregnant sows versus non-pregnant sows. The numbers equal to 1.00 means the expression level doesn't change between the two groups. The numbers >1.00 means the expression level is increased in pregnant sows while numbers <−1.00 indicate that the expression level is reduced in pregnant sows. The numbers with “*” are statistically significant (*P*<0.05), and which with“**” are statistically very significant (*P*<0.01).

## Discussion

As we all know, early and accurate diagnosis of non-pregnant sows will contribute to increase reproductive efficiency and financial income in pig production by reducing nonproductive days (NPD) per sow per year [21]. However, most of the currently available methods for sow early diagnosis are effective after at least 21 days of gestation [22]. In addition to using these routine methods for pregnancy diagnosis in swine, the maternal peripheral blood of early pregnant sows may contain some molecular markers. To identify molecular markers of sow early pregnancy in maternal peripheral blood, mRNA expression profiles of peripheral blood samples from pregnant sows were compared to those of control samples (peripheral blood samples from non-pregnant sows) at day 14 post-insemination. In the present study, we successfully identified 183 genes that were significantly (*P*<0.05) differentially expressed in the maternal peripheral blood between pregnant and non-pregnant sows (Table S1 and S2).

Of the three significantly enriched GO terms (Table 2), the G protein-coupled receptor (GPCR) signaling pathway plays an essential role in signal transmission and perception of and response to the environment [23], and may be involved in pregnancy recognition in humans and animals. Among the seven members involved in the biological process of GPCR signaling pathway, LPAR3 (Lysophosphatidic acid receptor 3) is one member of G-protein coupled receptors LPAR1-LPAR4, and is considered to be an uterine receptivity marker critical for embryo migration and spacing in mice [24]. Deletion of *LPAR3* gene in mice causes breed-related disorders including delayed implantation [25]. *RXFP4* (Relaxin/insulin-like family peptide receptor 4) is one of the cognitive receptors of relaxin-3 [26]. The expression of this gene is found to be increased during early pregnancy in maternal peripheral blood of sows. Although the physiological significance of GALP (galanin-like peptide) in pigs have not been elucidated, we suppose that it has the similar function, stimulating LH release, as observed in female mice [27]. In pigs, LH pulses can provoke prostaglandin secretion from endometrium during early pregnancy [28].

The only one significantly enriched pathway, Arachidonic acid metabolism (containing differently expressed gene *CBR1, CBR2* and *GPX6*), may be involved in early pregnancy by altering production of prostaglandins. The deduction is based on the following facts: First, arachidonic acid is the precursor molecule for prostaglandin E2 (PGE_2_) synthesis [29]; then CBR1 (carbonyl reductase 1, also referred to PG 9-ketoreductase) can convert PGE_2_ into PGF_2α_ [24], these two prostaglandins have pleiotropic effects during sow pregnancy, potentially involving in embryo development, ovarian function and maternal recognition of pregnancy [30]. Second, although the exact function of CBR2 in pig is not clear yet. Zhang *et al.* suggested that CBR2, together with other enzymes such as PTGS1/2, PTGES/S2 and CBR1, plays a critical role in the PG synthesis [31]. Third, *GPX6* is one member of the GPx family, which contains 6 antioxidant glutathione peroxidases (GPxs) and plays important roles in mammalian reproduction and pregnancy [32]. Of note, although the expression of this gene is reported to be restricted to embryos and adult olfactory epithelium in mammals [33], we detected its mRNA expression in the peripheral blood of sows.

For the remaining differently expressed genes listed in Table 3, *USP18* (Ubiquitin specific peptidase 18) has been identified as an early marker of conceptus development or uterine receptivity in cows [34], [35]. The up-regulation of LHB, which encodes the beta subunit of luteinizing hormone (LH), has also been observed in early pregnant mares. The possible role of the up-regulation of LHB is to increase release of bioactive LH into the uterine environment during early pregnancy, and the latter exert paracrine effects preparing the uterus for conceptus implantation [36]. In vertebrates, the orphan nuclear receptor steroidogenic factor 1 (SF-1, officially designated NR5A1) participates in the regulation of genes in reproductive endocrine signaling axes [37] and ovarian function [38].

In conclusion, our study provided novel insights into discovering specifically expressed genes at the initial stage of implantation in peripheral blood in pigs. The down-expressed *CBR1* and up-regulated *CBR2* in peripheral blood at day 14 of pregnant sows might contribute to the change of PGE_2_/PGF_2α_ ratio during early implantation. The results implied that these differentially expressed genes and the related pathways in peripheral blood at day 14 post-insemination could be potential biomarkers to identify porcine pregnancy establishment. One limitation of the present study is we are not sure if there is circulating fetal nucleic acid in sow maternal peripheral blood.

In summary, it is the first global microarray based study investigating differentially regulated genes in maternal peripheral blood of pregnant versus non-pregnant sows at the early stage of implantation. We identified several early pregnancy-specific differentially expressed genes. Some of these genes participate in the biological process of G protein-coupled receptor (GPCR) signaling pathway and/or KEGG pathway of arachidonic acid metabolism. This finding makes it intriguing to speculate that the early pregnancy-specific markers shown in maternal peripheral blood of pregnant sows can facilitate early pregnancy diagnosis in pigs.

## Supporting Information

Table S1 **Genes with higher mRNA levels in maternal peripheral blood of pregnant sows compared to non-pregnant sows.**
(XLS)

Table S2 **Genes with lower mRNA levels in maternal peripheral blood of pregnant sows compared to non-pregnant sows.**
(XLS)

File S1 **Total RNA fast extraction procedure.**
(DOC)
