# Supplementary material for: Comparative Transcriptome Analysis Reveals Early Pregnancy-Specific Genes Expressed in Peripheral Blood of Pregnant Sows
Source: PLoS One. 2014 Dec 5;9(12):e114036. doi: 10.1371/journal.pone.0114036 (PMC4257664; doi:10.1371/journal.pone.0114036)
Supplement: File S1 — Total RNA fast extraction procedure. (DOC) [file pone.0114036.s003.doc]

Total RNA fast extraction procedure (Bioteke, Cat No. RP4001):

1. Pipet 750μl Lysis Buffer RLS to a 1.5ml microcentrifuge tube. Add 250μl whole blood to the Lysis Buffer RLS in the microcentrifuge tube. Vortex for 2min to mix thoroughly.

2. Incubate for 10 min at RT.

3. Add 150μl chloroform and shake tubes vigorously for 15 sec, and incubate for 3 min at RT.

4. Centrifuge the samples at 12,000 rpm for 10min at 4°C. The mixture separates into 3 phases: an upper aqueous phase, interphase and a lower phenol-chloroform phase. RNA remains in the upper aqueous phase. The volume of aqueous phase is around 60% of Lysis Buffer RLS for homogenization.

5. Transfer the aqueous phase to a fresh tube, add 500μl 70% ethanol.

6. Place the Spin-column AC to the Collection Tube, transfer the alcohol-aqueous mixture to the Spin-column AC, centrifuge at 10,000 rpm for 1 min, and discard the filtrate.

7. Place the Spin-column AC back to Collection Tube; add 500μl Protein Precipitation Buffer RE to Spin-column AC, centrifuge at 10,000 rpm for 1min, and discard the filtrate.

8. Place the Spin-column AC back on the Collection Tube, add 700 μl Washing Buffer RW, centrifuge at 12,000 rpm for 1 min, and discard the filtrate.

9. Add 500 μl Washing Buffer RW, centrifuge at 12,000 rpm for 1min, and discard the filtrate.

10. Place the Spin-column AC to the Collection Tube and spin for 2min to remove the residual fluid.

11. For the resolution of RNAs, place the Spin-column AC to a 1.5ml RNase-free centrifuge tube, add 50-80μl RNase-free ddH_2_O.
